# Transfer of Respiratory Syncytial Virus Prefusion F Protein Antibody in Low Birthweight Infants

**DOI:** 10.1093/ofid/ofae314

**Published:** 2024-07-22

**Authors:** Alisa B Kachikis, Kalee Rumfelt, Mindy Pike, Monica Sosa, Jennifer E Stolarczuk, Hye Cho, Linda O Eckert, Emily T Martin, Janet A Englund

**Affiliations:** Department of Obstetrics and Gynecology, University of Washington, Seattle, Washington, USA; Department of Epidemiology, University of Michigan School of Public Health, Ann Arbor, Michigan, USA; Department of Obstetrics and Gynecology, University of Washington, Seattle, Washington, USA; Department of Obstetrics and Gynecology, University of Washington, Seattle, Washington, USA; Department of Obstetrics and Gynecology, University of Washington, Seattle, Washington, USA; School of Medicine, SUNY Upstate Medical University, Syracuse, New York, USA; Department of Obstetrics and Gynecology, University of Washington, Seattle, Washington, USA; Department of Global Health, University of Washington, Seattle, Washington, USA; Department of Epidemiology, University of Michigan School of Public Health, Ann Arbor, Michigan, USA; Department of Pediatrics, Seattle Children's Hospital Research Institute, Seattle, Washington, USA; Department of Pediatrics, University of Washington, Seattle, Washington, USA

**Keywords:** high-risk pregnancies, low birthweight infants, respiratory syncytial virus, RSV prefusion F protein antibody, transplacental antibody transfer

## Abstract

**Background:**

Respiratory syncytial virus (RSV)–associated lower respiratory tract infection contributes significantly to morbidity/mortality worldwide in low birthweight (LBW) infants (<2500 g). Studies have demonstrated decreased maternal immunoglobulin G (IgG) transfer of various antibodies to LBW infants. We aimed to evaluate naturally acquired RSV anti–prefusion F protein (anti-preF) antibody transfer in pregnancies with LBW versus normal birthweight (NBW) infants.

**Methods:**

In this cohort study conducted among pregnant individuals and their infants, we tested paired maternal and singleton infant cord samples for RSV anti-preF IgG via an electrochemiluminescence immunoassay, using linear regression to evaluate associations between LBW and anti-preF IgG. Covariates included seasonality, insurance, small-for-gestational-age birthweight, and gestational age at delivery.

**Results:**

We tested maternal/cord RSV anti-preF IgG from 54 and 110 pregnancies with LBW and NBW infants, respectively. Of LBW infants, 22 (40.7%) were born both preterm and with small-for-gestational-age birthweight. The median (interquartile range) gestational age at delivery and birthweight were 34.0 (31.7–37.1) weeks and 1902 (1393–2276) g for LBW infants versus 39.1 (38.3–39.9) weeks and 3323 (3109–3565) g for NBW infants (both *P* < .001). In unadjusted comparisons, preterm infants had significantly lower cord anti-preF IgG levels and cord-maternal IgG ratios compared with full-term infants, while LBW infants had significantly lower cord-maternal IgG ratios than NBW infants (all *P* < .01). After adjustment for covariates, there was no difference in cord-maternal IgG ratios (β =−0.29 [95% confidence interval, −.63 to .05]) between LBW and NBW infants.

**Conclusions:**

We documented robust transfer of maternal RSV anti-preF IgG in pregnancies with both LBW and NBW infants. Further studies are needed to assess immune protection in at-risk infants.

## Teaser

Robust, efficient transplacental transfer of respiratory syncytial virus anti–prefusion F protein immunoglobulin G occurs in pregnancies with normal- and lowbirthweight infants. Prematurity, but not low birth weight, resulted in lower transplacental transfer and cord concentrations of naturally acquired antibody.

Respiratory syncytial virus (RSV)–associated lower respiratory tract infection contributes significantly to infant morbidity and mortality worldwide [1, 2]. Infants with low birthweight (LBW), defined as <2500 g, such as those born prematurely (<37 weeks’ gestational age [GA]) or with birthweight small for GA (SGA; <10^th^ percentile for GA) are especially at risk for serious sequelae from common respiratory viruses including RSV [3–6]. Maternal immunization, the transfer of maternal antibody across the placenta, is an important mechanism for prevention of serious infections in early infancy [7, 8]. In 2012, boosters against pertussis were introduced during pregnancy, resulting in decreased rates of illness and death due to pertussis in infants [9, 10]. Similar decreases in infant respiratory illness have been seen following administration of influenza vaccine and, more recently, with severe acute respiratory syndrome coronavirus 2 vaccines during pregnancy [11, 12].

It is well established that transplacental transfer of maternal immunoglobulin G (IgG) increases throughout gestation [13, 14]. Other factors may also play a role in decreasing transfer, including IgG subclass, maternal hypergammaglobulinemia, infant birthweight, and maternal comorbid conditions, especially those affecting the placenta [13, 15, 16]. The degree to which these factors combine to affect transfer, and the clinical implications of resulting decreases, can be difficult to measure based on GA alone. Prior studies on transplacental IgG transfer in The Gambia and Sri Lanka, for example, have demonstrated decreased IgG transfer in pregnancies with both preterm infants and LBW infants [15, 17]; however, the reason for this decreased transfer has not been fully ascertained. Since LBW encompasses infants born preterm as well as both preterm and full-term infants with SGA birthweight, pregnancy conditions that often result in SGA birthweight, such as placental insufficiency, may contribute to decreased transplacental antibody transfer. Given the increased morbidity and mortality rates associated with respiratory pathogens of LBW infants, particularly in low- and middle-income countries (LMICs). where LBW is estimated to contribute to about 75% of perinatal deaths [18, 19], it is important to understand the implications for these at-risk infants in the context of widescale maternal vaccine implementation.

A maternal RSV vaccine has recently been approved in the United States, for administration during pregnancy at 32–36 weeks’ GA to boost infant immune protection against RSV [20–22]. Given the approval of the maternal RSV vaccine, the anticipated rollout of this vaccine in all resource settings globally, and the potential clinical ramifications, we aimed to evaluate RSV anti–prefusion F protein (anti-preF) antibody transfer in LBW versus normal-birthweight (NBW) infants in pregnancies with naturally acquired maternal RSV immunity.

## METHODS

### Participants

We recruited participants as part of an ongoing prospective cohort study on maternal immunizations in low- and high-risk pregnancies at the University of Washington (UW), which started in 2018. Maternal and cord blood samples were collected at the time of delivery after receipt of written informed consent. We selected all participants with an SGA infant from 2018 to 2021 with paired maternal and cord blood samples. A convenience sample of participants with non-SGA infants from 2018–2021 with paired maternal and cord serum samples matched by GA at birth was obtained, at a ratio of approximately 1 SGA to 2 non-SGA birthweight infants. Exclusion criteria were a multiple (eg, twin) pregnancy or a known fetal genetic anomaly. Of 262 pairs from the cohort, 187 had samples sent for antibody testing. Of these, a total of 164 pairs met inclusion criteria and were included in the current analysis. This study was reviewed and received ethical approval through the Seattle Children's Hospital Institutional Review Board and the UW Human Subjects Division.

### Variables

We abstracted and entered clinical data of participants from the electronic medical record (EMR) as well as linked Washington State Immunization Registry data into a REDCap (version 12.1.1; 2022) database. To report diversity among our study population, we collected self-reported race and ethnicity data from the EMR and categorized these variables based on the Centers for Disease Control and Prevention's categories [23]. Insurance status categories included public, private, Tricare (military), federal, and other. Body mass index (BMI) was based on maternal weight at the time of delivery.

We designated participants with type 1 or type 2 diabetes mellitus as having pregestational diabetes and participants with a diagnosis of hypertension before 20 weeks’ GA as having chronic hypertension. We categorized participants as having preeclampsia if they had a diagnosis of preeclampsia without severe features, preeclampsia with severe features, chronic hypertension with superimposed preeclampsia, or eclampsia based on American College of Obstetricians and Gynecologists criteria [24]. Participants with autoimmune or inflammatory conditions such as systemic lupus erythematous or Crohn disease were designated as having autoimmune or inflammatory conditions. Participants were designated as being on immunosuppressive medications if they received long-term corticosteroids, biologics, or other immunosuppressants during pregnancy. We calculated infant birthweight percentiles based on Olsen curves [9, 10], which were defined as appropriate for GA (AGA) if at or above the 10th percentile and SGA if below the 10th percentile for GA. We determined seasonality based on the birth quarter of infants’ birth dates.

### Antibody Testing

Maternal blood was collected within 72 hours of delivery, and cord blood was collected at delivery. All serum samples were stored at −80°Ce after centrifugation at 1800 revolutions per minute for 20 minutes. Maternal total IgG was tested in the UW Immunology Clinical Laboratory using an Optilite analyzer with standard reagents. Maternal and cord blood samples were tested for RSV anti-preF IgG antibody using an electrochemiluminescence immunoassay, also known as the Meso Scale Discovery (MSD) assay. RSV preF antigen was provided to MSD by AstraZeneca. Serum samples were diluted to dilutions of 1:5000 and 1:25 000 and processed following the MSD protocol insert [25]. Briefly, diluted serum, calibrators, and controls were added to MSD respiratory panel 1 IgG plates and incubated at room temperature for 2 hours. Plates were washed, monoclonal SULFO-TAG–labeled anti-human specific IgG antibody detection solution was added, and plates were incubated for 1 hour at room temperature. After plates were washed, antibody detection solution was added to the plates, and electrochemiluminescence was measured in relative light units using the MSD SECTOR S600 plate reader. Sample antibody concentrations were calculated by plotting their relative light unit outputs onto the standard curve generated from serially diluted calibrators that were run on the same plate. We reported results as arbitrary units per milliliter and log_2_-transformed antibody levels for final analysis.

### Analyses

Baseline demographic and pregnancy characteristics are reported as absolute numbers and percentages or medians and interquartile ranges. We compared these variables using *t*, χ^2^, and Fisher exact tests. We evaluated the relationship between birthweight category (LBW vs NBW) and maternal and cord RSV anti-preF IgG levels using Wilcoxon rank sum tests and linear regression analyses. Similar analyses were performed for ratios of cord to maternal anti-preF IgG, which we tested with *t* tests, Pearson correlation, and linear regression. Generally, cord-maternal antibody ratios ≥1 are considered to indicate “efficient” transfer of IgG across the placenta [7].

Covariates were selected a priori and based on significant associations between the exposure of LBW and the outcomes of cord anti-preF IgG. The first minimally adjusted linear regression model included seasonality of the delivery date. A second minimally adjusted linear regression model included seasonality and insurance status. The first fully adjusted linear regression model included seasonality, insurance status, and SGA. A second fully adjusted model adjusted for GA at delivery instead of SGA. We performed statistical analyses using Stata (version 18.0; StataCorp) [26] and SAS (version 9.4 (SAS Institute) software [27] and considered a 2-sided *P* value <.05 to be statistically significant. We followed Strengthening the Reporting of Observational Studies in Epidemiology (STROBE) reporting guidelines [28].

## RESULTS

### Baseline Characteristics

Between May 2018 and July 2021, a total of 164 pregnancies met inclusion criteria, of which 54 (32.9%) resulted in LBW and 110 (67.1%) in NBW infants (Table 1). There were no differences in racial or ethnic identities between participants with LBW infants and those with NBW infants, although fewer participants in the LBW group had private insurance (*P* = .02). Groups were also similar in terms of maternal comorbid conditions, although participants with LBW infants were more likely to have chronic hypertension (*P* = .002) and/or have preeclampsia diagnosed (*P* < .001) during their pregnancy. There was no difference in influenza vaccination rates between the groups, although participants with LBW infants were less likely to receive the tetanus, diphtheria, and acellular pertussis (Tdap) vaccine (*P* = .001).

**Table 1.** Baseline Characteristics and Pregnancy Outcomes

Characteristic or Outcome	Pregnancies, No. (%)^a^			*P* Value^b^
	Total (n = 164)	LBW (n = 54)	NBW (n = 110)	
Baseline maternal characteristics				
Enrollment year				
2018	32 (19.5)	16 (29.6)	16 (14.5)	<.001
2019	22 (13.4)	14 (25.9)	8 (7.3)	
2020	67 (40.9)	14 (25.9)	53 (48.2)	
2021	43 (26.2)	10 (18.6)	33 (30.0)	
Maternal age, median (IQR), y	33 (29–37)	31 (28–36)	33 (30–37)	.11
Gravidity, median (IQR)	2 (1–3)	2 (1–3)	2 (1–3)	.40
Parity, median (IQR)	0 (0–1)	0 (0–1)	1 (0–1)	.93
Race				
American Indian or Alaska Native	1 (0.7)	1 (2.0)	0 (0.0)	.08
Pacific Islander	4 (2.5)	3 (5.9)	1 (1.0)	
Asian	27 (17.4)	7 (13.7)	20 (19.2)	
Black or African American	11 (7.1)	6 (11.7)	5 (4.8)	
White	111 (71.6)	34 (66.7)	77 (74.0)	
Other	1 (0.7)	0 (0.0)	1 (1.0)	
Hispanic ethnicity	15 (9.6)	3 (5.9)	12 (11.3)	.39
Insurance status				
Public	30 (18.3)	15 (27.8)	15 (13.7)	.02
Private	125 (76.2)	34 (62.9)	91 (82.7)	
Tricare/federal/other	9 (5.5)	5 (9.3)	4 (3.6)	
BMI at delivery, median (IQR)^c^	30.8 (27.0–33.9)	30.5 (26.4–33.8)	31.0 (27.5–34.0)	.17
Pregestational DM	4 (2.4)	3 (5.6)	1 (0.9)	.11
Preeclampsia	22 (13.4)	17 (31.5)	5 (4.6)	<.001
Chronic HTN	18 (11.0)	12 (22.2)	6 (5.5)	.002
Autoimmune/inflammatory disorder	6 (3.7)	3 (5.6)	3 (2.7)	.40
Immunosuppressing meds	8 (4.9)	2 (3.7)	6 (5.5)	.48
Receipt of influenza vaccine during pregnancy	114 (69.5)	37 (68.5)	77 (70.0)	.86
Receipt of Tdap vaccine during pregnancy	153 (93.3)	45 (83.3)	108 (98.2)	.001
Pregnancy outcomes				
Birth timing (seasonality)				
Q1	44 (26.8)	24 (44.4)	20 (18.2)	<.001
Q2	34 (20.7)	14 (25.9)	20 (18.2)	
Q3	49 (29.9)	10 (16.7)	40 (36.3)	
Q4	37 (22.6)	7 (13.0)	30 (27.3)	
GA at delivery, median (IQR), wk	38.6 (36.0–39.3)	34.0 (31.7–37.1)	39.1 (38.3–39.9)	<.001
GA range at delivery, wk	26.0–41.9	26.0–39.0	34.3–41.9	NA
Preterm delivery				
<34 0/7 wk	26 (15.9)	13 (24.1)	13 (11.8)	<.001
34 0/7–36 6/7 wk	26 (15.9)	26 (48.2)	0 (.0)	
Mode of delivery				
Vaginal	70 (42.7)	16 (29.6)	54 (49.1)	.02
Cesarean	94 (57.3)	39 (70.4)	56 (50.9)	
BW, median (IQR), g	3113 (2292–3429)	1902 (1393–2276)	3323 (3109–3565)	<.001
SGA (<10th percentile BW)	42 (25.6)	36 (66.7)	6 (5.5)	<.001
Preterm/SGA status				
Preterm and SGA	22 (13.4)	22 (40.7)	0	<.001
Preterm and AGA	30 (18.3)	17 (31.5)	13 (11.8)	
Full term and SGA	20 (12.2)	14 (25.9)	6 (5.5)	
Full-term and AGA	92 (56.1)	1 (1.9)	91 (82.7)	
Sex of infant				
Female	85 (51.8)	26 (48.2)	59 (53.6)	.51
Male	79 (48.2)	28 (51.8)	51 (46.4)	
NICU admission	54 (33.5)	39 (75.0)	15 (13.8)	<.001
Maternal total IgG at delivery, median (IQR), mg/dL	716 (603–897)	770 (603–953)	692 (600–889)	.40

Abbreviations: AGA, appropriate for GA; BMI, body mass index; BW, birthweight; DM, diabetes mellitus; GA, gestational age; HTN, hypertension; IgG, immunoglobulin G; IQR, interquartile range; LBW, low BW; NA, not available; NBW, normal BW; NICU, neonatal intensive care unit; Q1 (etc), quarter 1 (etc); SGA, small for GA; Tdap, tetanus, diphtheria, and acellular pertussis vaccine.

^a^Data represent no. (%) of pregnancies unless otherwise specified.

^b^Continuous variables were compared using *t* tests; categorical variables, using χ^2^ and Fisher exact tests.

^c^BMI calculated as weight in kilograms divided by height in meters squared.

### Pregnancy Outcomes

The majority of LBW infants were born in quarter 1 or 2, while in most NBW infants were born in quarter 3 or 4 (*P* < .001; Table 1). Pregnancies with LBW infants had lower GAs at delivery and lower birthweights than pregnancies with NBW infants (both *P* < .001). LBW infants included those born preterm (n = 39) and those who were SGA (n = 36); 22 (40.7%) were both born preterm and SGA (Table 1). Of LBW infants, 26 (48.2%) were delivered at <34 weeks’ GA. LBW infants were also more likely to be born via cesarean delivery (*P* = .02) and be admitted to the neonatal intensive care unit (*P* < .001). There were significantly more infants with SGA birthweight in the LBW group (n = 36 [66.7%]) than in the NBW group (n = 6 [5.5%]; *P* < .001).

### RSV Anti-preF IgG Analyses

Median RSV anti-preF IgG concentrations were higher in cord than in maternal samples regardless of birthweight status (Table 2). The median cord-maternal antibody transfer ratios all exceeded 1, with a median ratio of 1.63 (interquartile range, 1.18–2.01). Ratios were significantly higher in the NBW than in the LBW group (median, 1.71 [1.23–2.06] vs 1.37; [0.99–1.81], respectively; *P* = .002).

**Table 2.** Maternal and Cord Respiratory Syncytial Virus Anti–Prefusion F Protein Immunoglobulin G Levels and Cord-Maternal Ratios in the Total Cohort and by BirthWeight Status

RSV Anti-preF IgG Measure	Median Value (IQR) by BW Category			*P* Value^a^
	Total (n = 164)	LBW (n = 54)	NBW (n = 110)	
IgG level, AU/mL				
Maternal	86 454 (51 379–133 891)	97 590 (53 025–151 170)	84 643 (46 999–126 236)	.21
Cord	136 017 (82 253–214 474)	122 954 (70 465–199 905)	140 366 (86 192–215 556)	.42
Cord-maternal IgG ratio^b^	1.63 (1.18–2.01)	1.37 (0.99–1.81)	1.71 (1.23–2.06)	.002

Abbreviations: Anti-preF, anti–prefusion F protein; AU, arbitrary units; BW, birthweight; IgG, immunoglobulin G; IQR, interquartile range; LBW, low BW; NBW, normal BW; RSV, respiratory syncytial virus.

^a^Maternal and cord anti-spike IgG levels were compared using Wilcoxon rank sum tests; ratios, using *t* tests.

^b^Cord divided by maternal anti-preF IgG level.

**Figure 1.**
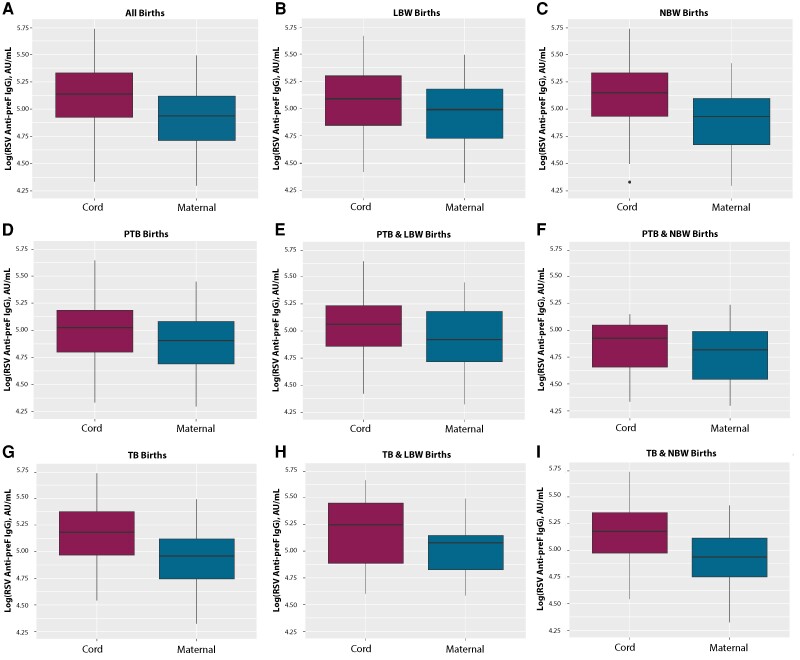
Box plots of cord and maternal respiratory syncytial virus (RSV) anti-prefusion F protein (anti-preF) immunoglobulin G (IgG) titers (in arbitrary units [AU] per milliliter) by birthweight category and gestational age (GA) at delivery. *A,* Entire cohort. *B,* All pregnancies with preterm deliveries (at <37 weeks’ GA). *C*, All pregnancies with full-term deliveries (at ≥37 weeks’ GA). *D*, All pregnancies with low birthweight (LBW) infants. *E*, Pregnancies with preterm deliveries and LBW infants. *F*, Pregnancies with full-term deliveries and LBW infants. *G,* All pregnancies with normal-birthweight (NBW) infants. *H*, Pregnancies with preterm deliveries and NBW infants. *I,* Pregnancies with full-term deliveries and NBW infants.

We also compared maternal and cord anti-preF RSV IgG levels as well as cord-maternal IgG ratios by GA at delivery (Table 3 and Figure 1) and birthweight status stratified by preterm and full-term deliveries (Tables 3 and 4). In our comparison of pregnancies with preterm versus full-term deliveries regardless of birthweight status, pregnancies with preterm deliveries had significantly lower cord anti-preF IgG levels (*P* = .001) and cord-maternal IgG ratios (*P* < .001) than pregnancies with full-term deliveries (Table 3 and Figure 1*A*–1*C*). There was no difference between pregnancies with LBW infants with preterm versus full-term deliveries in terms of maternal or cord anti-preF IgG levels or cord-maternal anti-preF IgG ratios (Table 3 and Figure 1*D*–1*F*). In contrast, we found significant differences in maternal anti-preF IgG levels (*P* = .04), cord anti-preF IgG levels, and cord-maternal anti-preF IgG ratios among preterm and full-term infants with NBW (both *P* < .001; Table 4 and Figure 1*G*–1*I*). Finally, we compared antibody concentrations by SGA and AGA birthweight percentiles in the total cohort (Supplementary Table S1) and stratified by LBW and NBW groups (Supplementary Tables S2 and S3), but antibody concentrations did not differ significantly between pregnancies with SGA and those with AGA birthweight infants in any of the comparisons.

**Table 3.** Maternal and Cord Respiratory Syncytial Virus Anti–Prefusion F Protein Immunoglobulin G Levels and Cord-Maternal Ratios by Gestational Age at Delivery in the Total Cohort and in Low Birthweight Infants

RSV Anti-preF IgG Measure	Median Value (IQR) by GA at Delivery				*P* Value^a^
	All GAs	<34 wk	≥34 to <37 wk	≥37 wk	
Total cohort	n = 164	n = 26	n = 26	n = 112	…
IgG level, AU/mL					
Maternal	86 454 (51 379–133 891)	97 590 (55 247–154 543)	67 057 (39 618–99 197)	90 290 (55 398–133 891)	.09
Cord	136 017 (82 253–214 474)	107 589 (70 465–177 962)	95 921 (45 548–139 984)	152 231 (91 145–238 965)	.001
Cord-maternal IgG ratio^b^	1.63 (1.18–2.01)	1.20 (0.88–1.67)	1.14 (1.00–1.64)	1.72 (1.33–2.09)	<.001
LBW infants	n = 54	n = 26	n = 13	n = 15	…
IgG level, AU/mL					
Maternal	97 590 (53 025–151 170)	97 590 (55 247–154 543)	69 117 (49 443–100 718)	119 496 (54 742–151 170)	.31
Cord	122 954 (70 465–199 905)	107 589 (70 465–177 962)	116 725 (50 266–164 742)	175 764 (70 706–317 985)	.27
Cord-maternal IgG ratio^b^	1.37 (0.99–1.81)	1.20 (0.88–1.67)	1.30 (0.98–1.76)	1.50 (1.23–1.99)	.22

Abbreviations: Anti-preF, anti–prefusion F protein; AU, arbitrary units; GA, gestational age; IgG, immunoglobulin G; IQR, interquartile range; LBW, low birthweight; RSV, respiratory syncytial virus.

^a^Maternal and cord anti-spike IgG levels were compared using Wilcoxon rank sum tests; ratios, using *t* tests.

^b^Cord divided by maternal anti-preF IgG level.

**Table 4.** Maternal and Cord Respiratory Syncytial Virus Anti–Prefusion F Protein Immunoglobulin G Levels and Cord-Maternal Ratios by Gestational Age at Delivery in Normal-BirthWeight Infants

RSV Anti-preF IgG Measure	Median Value (IQR) by GA at Delivery in NBW Infants			*P* Value^a^
	All GAs (n = 110)	34–37 wk (n = 13)	>37 wk (n = 97)	
IgG level, AU/mL				
Maternal	84 643 (46 999–126 236)	66 720 (35 027–97 720)	86 200 (56 055–131 140)	.04
Cord	140 366 (86 192–215 556)	84 127 (45 550–112 113)	149 130 (93 478–234 937)	<.001
Cord-maternal IgG ratio^b^	1.71 (1.23–2.06)	1.13 (1.07–1.43)	1.76 (1.33–2.10)	<.001

Abbreviations: Anti-preF, anti-prefusion F protein; AU, arbitrary units; IgG, immunoglobulin G; IQR, interquartile range; NBW, normal-birthweight; RSV, respiratory syncytial virus.

^a^Maternal and cord anti-spike IgG levels were compared using Wilcoxon rank sum tests; ratios, using *t* tests.

^b^Cord divided by maternal anti-preF IgG level.

**Figure 2.**
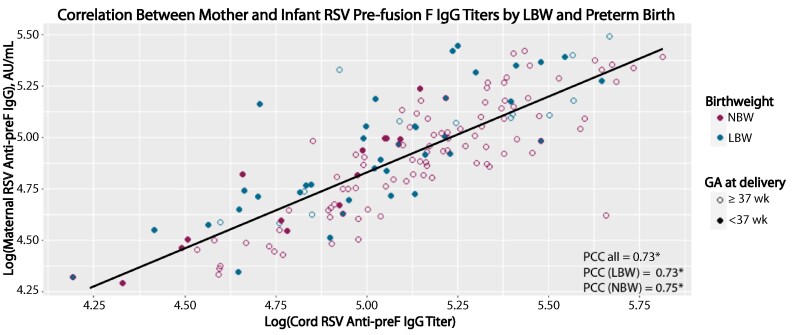
Correlation between maternal and cord respiratory syncytial virus (RSV) anti–prefusion F (anti-preF) protein immunoglobulin G (IgG) titers (in arbitrary units [AU] per milliliter) by birthweight category (normal birthweight [NBW] vs low birthweight [LBW]) and gestational age (GA) at delivery (preterm [GA <37 weeks] vs full-term [GA ≥37 weeks]). Visualization of correlations between maternal and infant log adjusted RSV anti-preF IgG across birthweight status and prematurity. Pearson correlation coefficients (PCCs) were calculated using untransformed RSV anti-preF IgG titers. *P < .001.

Pearson correlation coefficients showed strong statistically significant correlations between maternal and cord RSV anti-preF IgG levels for the entire sample (Pearson correlation coefficient = 0.72; *P* < .001) and when stratified into LBW (0.73; *P* < .001) and NBW (0.75; *P* < .001) (Figure 2). There were weak associations between LBW and both maternal and cord RSV anti-preF IgG concentrations and while not statistically significant, they were similar across all models (Table 5).

**Table 5.** Association Between Low BirthWeight and Maternal and Cord Respiratory Syncytial Virus Anti–Prefusion F Protein Immunoglobulin G Levels and Cord-Maternal Ratios

RSV Anti-preF IgG Measure by BW	β Coefficient (95% CI)	*P* Value	Adjusted β Coefficient (95% CI)^a^	*P* Value	Adjusted β Coefficient (95% CI)^b^	*P* Value	Adjusted β Coefficient (95% CI)^c^	*P* Value	Adjusted β Coefficient (95% CI)^d^	*P* Value
Maternal IgG level										
NBW	0.0 (Ref)	.19	0.0 (Ref)	.37	0.0 (Ref)	.38	0.0 (Ref)	.27	0.0 (Ref)	.11
LBW	0.06 (−.03 to .16)		0.05 (−.05 to .15)		0.05 (−.06 to .15)		0.08 (−.06 to .21)		0.11 (−.03 to .24)	
Cord IgG level										
NBW	0.0 (Ref)	.41	0.0 (Ref)	.38	0.0 (Ref)	.56	0.0 (Ref)	.61	0.0 (Ref)	.10
LBW	−0.04 (−.15 to .06)		−0.05 (−.16 to .06)		−0.03 (−.15 to .08)		−0.04 (−.19 to .11)		0.12 (−.02 to .27)	
Cord-maternal IgG ratio										
NBW	0.0 (Ref)	.01	0.0 (Ref)	.04	0.0 (Ref)	.09	0.0 (Ref)	.12	0.0 (Ref)	.99
LBW	−0.39 (−.70 to −.086)		−0.34 (−.67 to −.01)		−0.29 (−.63 to .05)		−0.35 (−.79 to .09)		0.003 (−.643 to .44)	

Abbreviations: Anti-preF, anti–prefusion F protein; CI, confidence interval; IgG, immunoglobulin; LBW, low birthweight; NBW, normal birthweight; Ref, reference; RSV, respiratory syncytial virus.

^a^Adjusted for seasonality.

^b^Adjusted for seasonality and private health insurance.

^c^Adjusted for seasonality, private health insurance, and small-for-gestational-age birthweight.

^d^Adjusted for seasonality, private health insurance, and gestational age at delivery.

In our linear regression analyses, a moderate statistically significant association between LBW and lower cord-maternal antibody transfer ratio was produced for the unadjusted model (β = −0.39; *P* = .01) (Table 5) and this association remained significant after adjusting only for seasonality of birth (β = −0.34; *P* = .04). However, after adjustment for insurance status alone or with SGA birthweight or GA at birth in our fully adjusted models, there was no association between LBW and cord-maternal antibody transfer ratio (Table 5).

## DISCUSSION

Our cohort study found robust and efficient transfer of RSV anti-preF IgG across the placenta in pregnancies with NBW and LBW infants born in the United States but found no difference between the groups. In a first subanalysis of anti-preF IgG concentrations by preterm or full-term GA at delivery, we found significant differences between groups, but we did not see differences between groups in a second subanalysis by SGA and AGA birthweight. In addition, the median cord-maternal anti-preF IgG ratio was >1.5 for the entire cohort, which correlates well with findings from other published studies on transplacental transfer of RSV-specific antibodies [29, 30]. Published results of the Pfizer phase 3 clinical trial of the maternal RSV vaccine do not report cord-maternal antibody ratios, and the vast majority of participants delivered at full term (94.7%) [21]; however, based on GA at delivery and efficacy data, it can be assumed that transfer ratios were efficient for the anti-preF antibody generated by the maternal RSV vaccine [21]. While our study focuses on transfer of naturally acquired RSV-specific immunity, increasing infant protection via maternal RSV vaccine appears promising.

Our study investigates differences in transfer of RSV-specific immunity between pregnancies with LBW infants and those with NBW infants and found no differences in maternal or cord RSV anti-preF IgG concentrations between the groups with or without adjustment for covariates. Past studies on differences in RSV-specific antibody transfer between groups with different birthweights have mixed results with investigation of different specific antibodies or antibody subclasses. Okoko et al [15] found impaired placental transfer of IgG subclasses in LBW compared to NBW neonates in The Gambia. In additional studies in The Gambia and Sri Lanka, LBW was found to be independently associated with lower transfer of antibodies against measles virus, RSV, herpes simplex virus type 1, varicella-zoster virus, tetanus toxoid, and diphtheria toxoid [31, 32]. In contrast, studies from Bangladesh and Nepal did not find these differences in the context of RSV-specific antibodies [29, 33]. Notably, most studies conducted in LMICs do not differentiate SGA from prematurity when analyzing antibody transfer in LBW infants.

These conflicting findings may be due to differences in methods and standards for birthweight measures, estimation of GA, or confounding underlying comorbid conditions contributing to LBW [16]. Differences in findings may also be due to make up of cohorts (ie, the percentage of preterm infants included as well as the degree of prematurity in the cohort) and antibody-specific transfer kinetics. In our study, for example, only 15.8% of pregnancies were delivered at <34 weeks’ GA. GA is often difficult to measure, and accuracy depends on resource setting (eg, LMICs), including cultural norms regarding timing of antenatal care initiation, availability of technology, and differentiation between fetal age and growth anomalies [34]. In contrast, LBW is an objective infant weight measurement and, while potentially encompassing both preterm and SGA birthweight infants, is a more feasible and reproducible measurement to obtain in most global resource settings. Focusing on birthweight, a truly objective measure, eliminates issues related to GA that may be particularly present in studies in LMICs, with later entry to antenatal care or lack of available ultrasonography. While our study did not find differences in transplacental transfer of RSV anti-preF protein among pregnancies with LBW and those with NBW infants, further research in this area is needed.

By contrast, when we stratified our analyses by delivery GA rather than birthweight criteria, we found significant differences in cord anti-preF concentrations as well as cord-maternal anti-preF IgG ratios in both the entire cohort and the NBW group only. In contrast, results of our subanalyses of anti-preF concentrations by SGA and AGA birthweight percentiles were not significant. Our findings raise the question of whether differences in cord IgG levels and cord-maternal IgG ratios seen in LBW infants in prior studies are actually driven by lower GA at delivery (ie, prematurity) rather than SGA birthweight (ie, placental insufficiency) or other factors, such as IgG subclass [15].

In their studies on RSV-specific IgG transplacental transfer in Bangladesh and Nepal, Chu et al [29, 33] did not find differences in IgG transfer ratios among pregnancies with SGA versus AGA infants. For both studies, it is important to note that there were rigorous protocols for participant inclusion and exclusion criteria and variables related to determining GA. The data from the study conducted in Bangladesh were obtained as part of a randomized prospective controlled trial of influenza vaccine in pregnancy [35]. In Nepal, GA was prospectively determined based on last menstrual period and a house-to-house census of married women every 5 weeks for the duration of the study's enrollment period [33].

Similarly, in our study, GA was determined based on American College of Obstetricians and Gynecologists criteria [36], with the estimated date of delivery for the majority of participants determined by last menstrual period and first-trimester ultrasound or by first-trimester ultrasound alone. In studies conducted in different resource settings or with variable rigor in determining GA, differences in cord IgG titers or cord-maternal IgG ratios seen in pregnancies with LBW infants may be driven more by prematurity than by SGA birthweight. Since LBW infants include preterm infants as well as infants with SGA birthweight, differences in cord IgG titers and cord-maternal IgG ratios may not be seen as readily as in our study, compared with differences in cord IgG titers and cord-maternal IgG ratios based on preterm versus full-term status in the entire cohort or in NBW pregnancies alone (Table 5). In addition, we found that cord-maternal anti-preF IgG ratios increased with increasing GA in our total cohort, which has been well documented in published literature.

Finally, our analyses showed that while lower cord-maternal RSV anti-preF antibody ratios were significantly associated with pregnancies with LBW infants in our unadjusted model or our mode minimally adjusted for seasonality, this association disappeared by with additional adjustment for insurance status. RSV seasonality varied throughout the years of our study, due in large part to the coronavirus disease 2019 pandemic and related factors, including such mitigation factors as masking and school closures [37, 38], and we cannot directly assess the impact of RSV epidemics in our study. However, in a classic study from Houston, Texas, in the United States, Glezen et al [39] found that the risk of infant RSV infection depended on the nature of the RSV season, the month of birth (ie, seasonality of birth month), and maternally derived cord anti-preF IgG levels.

Similarly, in a comparison of RSV-specific antibody transfer among mother-infant pairs in the Yukon-Kaskokwin Delta region of Alaska compared with a cohort from the Seattle area in the United States, Chu et al [40] found that while cord-maternal IgG ratios were generally efficient (>1) in both regions, ratios were significantly lower among those in rural Alaska than in the continental United States. Finally, Atwell et al [41] found associations in RSV-specific antibody transfer based on maternal hypergammaglobinulinemia, not associated with placental malaria as other studies had hypothesized. Interestingly, these studies indicate that, despite similar maternal RSV-specific IgG concentrations, differences in seasonality and cohort-intrinsic factors may affect the transfer of RSV-specific immunity. It remains to be seen whether these differences will still be apparent after introduction of the maternal RSV vaccine.

The current study had both strengths and limitations. Our study population was recruited based on stringent criteria from an institution that had many pregnancies with high- and low-risk conditions, and we were able to include a relatively large cohort of pregnancies with LBW and NBW infants. In addition, the EMR is robust, and variables related to LBW, and pregnancy and delivery outcomes could be extracted with high accuracy. Nonetheless, this was a convenience sample acquired over time at a single site; furthermore, the number of extremely preterm or very-low birthweight infants in our study, such as those delivered at <28 weeks’ GA or <1500 g, was very small. Our samples are from participants residing in a high-income country without comorbid conditions that may affect transplacental antibody transfer, such as malaria or human immunodeficiency virus (HIV) infections. While we were able to recruit appropriately sized cohorts of pregnancies with LBW and NBW, we were still limited by sample size, and there may be additional confounders that we were not able to consider. In addition, we report RSV anti-preF IgG levels based on the MSD assay, but we do not report results from neutralization assays; however, prior studies have shown good correlation between anti-preF IgG concentrations and the neutralizing activity of these antibodies [42, 43].

In conclusion, our study findings suggest that transplacental transfer of maternal RSV anti-preF antibody is robust and efficient, even in LBW infants. While there were differences in cord anti-preF antibody levels and cord-maternal IgG ratios between preterm and full-term deliveries, we did not find differences in pregnancies by birthweight category. The maternal RSV vaccine has promise to enhance RSV immunity and provide increased immune protection, regardless of seasonality or intrinsic cohort characteristics. Further research into transfer of RSV-specific immunity during pregnancy, especially in high-risk populations, is warranted to optimize protection of these at-risk infants.

## Supplementary Material

ofae314_Supplementary_Data

## Supplementary Data

Supplementary materials are available at *Open Forum Infectious Diseases* online. Consisting of data provided by the authors to benefit the reader, the posted materials are not copyedited and are the sole responsibility of the authors, so questions or comments should be addressed to the corresponding author.

## Notes

***Financial support*.** This project was supported by the National Institute of Allergy and Infectious Diseases (grant K23 AI153390), the Eunice Kennedy Shriver National Institute for Child Health and Human Development (a 2 K12H001264 Women’s Reproductive Health Research Award), the Thrasher Research Fund, and the National Center for Advancing Translational Sciences (grant UL1 TR002319).
