# Supplementary material for: Transfer of Respiratory Syncytial Virus Prefusion F Protein Antibody in Low Birthweight Infants
Source: Open Forum Infect Dis. 2024 Jul 22;11(7):ofae314. doi: 10.1093/ofid/ofae314 (PMC11261662; doi:10.1093/ofid/ofae314)
Supplement: ofae314_Supplementary_Data [file ofae314_supplementary_data.docx]

| **Supplemental Table.** Maternal and cord RSV anti-prefusion F (anti-preF) protein levels in AU/mL in the total cohort by SGA or AGA birthweight percentiles (**Table S1**) and stratified by SGA and AGA birthweight percentiles for LBW infants (**Table S2**) and NBW infants (**Table S3**) |
| --- |

| **Table S1.** Maternal and cord RSV anti-preF IgG results among all pregnancies with SGA and AGA birthweight infants | | | | |
| --- | --- | --- | --- | --- |
|  | Total (n=164) | | | p-value |
| Anti-preF IgG | Total | SGA (n=42) | AGA (n=122) |  |
| Maternal | 86,454 (51,379-133,891) | 86,476 (54,157-129,560) | 86,454 (47,336-136,642) | 0.83 |
| Cord | 136,017 (82,253-214,474) | 122,954 (68,279-248,751) | 136,785 (86,192-211,342) | 0.80 |
| Ratio | 1.63 (1.18-2.01) | 1.48 (1.05-1.94) | 1.65 (1.20-2.02) | 0.15 |

| **Table S2.** Maternal and cord RSV anti-preF IgG results among pregnancies with LBW infants stratified by SGA and AGA birthweights | | | | |
| --- | --- | --- | --- | --- |
|  | LBW (n=54) | | | p-value |
| Anti-preF IgG | Total | SGA (n=36) | AGA (n=18) |  |
| Maternal | 97,590 (53,025-151,170) | 87,821 (50,411-129,157) | 112,747 (70,674-207,014) | 0.23 |
| Cord | 122,954 (70,465-199,905) | 117,987 (66,474-250,254) | 135,582 (105,439-169,472) | 0.87 |
| Ratio | 1.37 (0.99-1.81) | 1.48 (1.03-1.88) | 1.21 (0.97-1.63) | 0.22 |

| **Table S3.** Maternal and cord RSV anti-preF IgG results among pregnancies with NBW infants stratified by preterm and full-term deliveries | | | | |
| --- | --- | --- | --- | --- |
|  | NBW (n=110) | | | p-Value |
| Anti-preF IgG | Total | SGA (n=6) | AGA (n=104) |  |
| Maternal | 84,643 (46,999-126,236) | 81,763 (72,070-138,178) | 84,643 (45,053-124,822) | 0.67 |
| Cord | 140,366 (86,192-215,556) | 188,731 (98,212-217,648) | 139,935 (85,160-215,016) | 0.54 |
| Ratio | 1.71 (1.23-2.06) | 1.52 (1.29-2.37) | 1.72 (1.23-2.04) | 0.97 |
| Notes: median (IQR); maternal and cord anti-spike IgG compared using Wilcoxon rank sum test; ratio is calculated as cord anti-preF IgG divided by maternal anti-preF IgG and compared using t-test  Abbreviations: AGA=appropriate for gestational age birthweight (>10^th^ percentile); anti-preF = anti-prefusion F protein; IgG = immunoglobulin G; LBW = low birthweight; NBW = normal birthweight; SGA=small for gestational age (<10^th^ percentile). | | | | |
